# Draft Genome Sequence of Uncultivated Toluene-Degrading *Desulfobulbaceae* Bacterium Tol-SR, Obtained by Stable Isotope Probing Using [^13^C_6_]Toluene

**DOI:** 10.1128/genomeA.01423-14

**Published:** 2015-01-15

**Authors:** Nidal Abu Laban, BoonFei Tan, Anh Dao, Julia Foght

**Affiliations:** Department of Biological Sciences, University of Alberta, Edmonton, Alberta, Canada

## Abstract

The draft genome of a member of the bacterial family *Desulfobulbaceae* (phylum *Deltaproteobacteria*) was assembled from the metagenome of a sulfidogenic [^13^C_6_]toluene-degrading enrichment culture. The “*Desulfobulbaceae* bacterium Tol-SR” genome is distinguished from related, previously sequenced genomes by suites of genes associated with anaerobic toluene metabolism, including *bss*, *bbs*, and *bam*.

## GENOME ANNOUNCEMENT

The deltaproteobacterial family *Desulfobulbaceae* comprises seven genera that reduce sulfate and/or sulfur (1). Of these, *Desulfocapsa*, *Desulfobulbus*, and *Desulfofustis* were recently distinguished by their ability to disproportionate inorganic sulfur compounds (2). *Desulfocapsa* is frequently detected in anaerobic hydrocarbon-impacted environments and is thought to play a role in anaerobic degradation of monoaromatics under different redox conditions (3–5). Recent DNA-stable isotope probing (DNA-SIP) of sulfidogenic microcosms and enrichment cultures revealed bacteria related to *Desulfocapsa* to be key toluene-degrading organisms (6–9), although few hydrocarbon-degrading *Desulfobulbaceae* have been cultivated.

To date, complete genome sequences are available for only four *Desulfobulbaceae*: *Desulfobulbus propionicus* DSM 2032^T^ (10), *Desulfocapsa sulfexigens* DSM 10523 (11), *Desulfotalea psychrophila* LSv54 (12), and *Desulfurivibrio alkaliphilus* AHT2 (ACYL01000000); plus eight draft *Desulfobulbus* genomes (http://patricbrc.org). However, genes specifically associated with anaerobic hydrocarbon activation have not been annotated in these genomes. Here, we provide the draft genome of an uncultivated *Desulfobulbaceae* member that is implicated in toluene degradation under sulfidogenic conditions and contains genes associated with anaerobic hydrocarbon metabolism.

The draft genome of this putative sulfate-reducing toluene degrader (herein named “*Desulfobulbaceae* bacterium Tol-SR”) was obtained by applying DNA-SIP to a [^13^C_6_]toluene-degrading, sulfate-reducing enrichment culture derived from oil sands tailings (N. Abu Laban et al., submitted for publication). Total DNA was isolated and fractionated by buoyant density ultracentrifugation, and DNA from the ^13^C-rich “heavy” fraction was sequenced using Illumina Mi-seq (http://tagc.med.ualberta.ca/). Metagenomic reads were subjected to quality control and *de novo* assembly using CLC Genomics Workbench (CLC-Bio, USA). Scaffolds associated with *Desulfobulbaceae* were binned from the metagenome using sequence composition- and homology-based methods and subjected to decontamination (13). The genomic bin was annotated using RAST (http://rast.nmpdr.org).

Phylogenetic analysis of the draft genome affiliated Tol-SR with the genus *Desulfocapsa*, but the single 16S rRNA gene had only 92% identity to *D. sulfexigens* DSM 10523 (CP003985), its closest cultivated match, thus precluding classification of Tol-SR to genus level. Furthermore, the average nucleotide identity with *D. sulfexigens* (NC_020304) and other published *Desulfobulbaceae* genomes (NC_014972 and NC_006138) was only 79% (using 1-kb fragment cut-off), indicating that Tol-SR is distinct from previously described *Desulfobulbaceae* (http://patricbrc.org).

The Tol-SR draft genome is ~4.2 Mb contained in 240 scaffolds (size range 1 to 193 kb) with 59% G+C content and contains 107 of 110 single copy genes (14), with best BLASTp hits to sulfate-reducing *Deltaproteobacteria*. Annotation in RAST revealed 40 RNA genes, and 4,036 protein-coding sequences were assigned to 354 SEED subsystem categories. In addition to the expected genes encoding catabolism, biosynthesis, and stress response, the draft genome includes genes encoding prophages, transposable elements and plasmids, and 15 genes for sulfur disproportionation and metabolism. Genes associated with anaerobic hydrocarbon activation were arranged in clusters, such as *bssABC* encoding subunits for benzylsuccinate synthase, a key enzyme for anaerobic activation of toluene by fumarate addition; *bbsBDEF* encoding beta-oxidation of benzylsuccinate; and *bamB-I* genes encoding components postulated to be involved in benzoyl-CoA dearomatization in anaerobes.

This draft genome broadens our understanding of the distribution of anaerobic hydrocarbon assimilation in *Desulfobulbaceae*.

### Nucleotide accession numbers.

The sequences from the whole-genome shotgun project investigating *Desulfobulbaceae* bacterium Tol-SR have been deposited at DDBJ/EMBL/GenBank under accession no. JROS00000000. The version described in this paper is the first version JROS01000000.
